# Candida glabrata Has No Enhancing Role in the Pathogenesis of *Candida*-Associated Denture Stomatitis in a Rat Model

**DOI:** 10.1128/mSphere.00191-19

**Published:** 2019-04-03

**Authors:** Junko Yano, Alika Yu, Paul L. Fidel, Mairi C. Noverr

**Affiliations:** a Department of Prosthodontics, Louisiana State University Health Sciences Center School of Dentistry, New Orleans, Louisiana, USA; b Department of Oral and Craniofacial Biology, Louisiana State University Health Sciences Center School of Dentistry, New Orleans, Louisiana, USA; Carnegie Mellon University

**Keywords:** *Candida albicans*, *Candida glabrata*, biofilms, candidiasis, host-pathogen interactions, mycology

## Abstract

Denture stomatitis (DS) is a condition characterized by inflammation of the oral mucosa in direct contact with dentures and affects a significant number of otherwise healthy denture wearers. *Candida-*associated DS is predominantly caused by Candida albicans, a dimorphic fungus that readily colonizes and forms biofilms on denture materials. Previous studies showed a requirement for *Candida* biofilm formation on both palate and dentures in infection and identified fungal morphogenic transcription factors, Efg1 and Bcr1, as key players in DS pathogenesis. While both C. albicans and Candida glabrata are frequently coisolated in mucosal candidiasis, a pathogenic role for C. glabrata in DS remains unknown. Using an established rat model of DS, we sought to determine whether C. glabrata alone or coinoculation with C. albicans establishes colonization and causes palatal tissue damage and inflammation. Rats fitted with custom dentures were inoculated with C. albicans and/or C. glabrata and monitored over a 4-week period for fungal burden (denture/palate), changes in body weight, and tissue damage via lactate dehydrogenase (LDH) release as well as palatal staining by hematoxylin and eosin (H&E) and immunohistochemistry for myeloperoxidase (MPO) as measures of inflammation. C. glabrata colonized the denture/palate similarly to C. albicans. In contrast to C. albicans, colonization by C. glabrata resulted in minimal changes in body weight, palatal LDH release, and MPO expression. Coinoculation with both species had no obvious modulation of C. albicans-mediated pathogenic effects. These data suggest that C. glabrata readily establishes colonization on denture and palate but has no apparent role for inducing/enhancing C. albicans pathogenesis in DS.

**IMPORTANCE** Many denture wearers suffer from *Candida*-associated denture stomatitis (DS), a fungal infection of the hard palate in contact with dentures. Biofilm formation by Candida albicans on denture/palate surfaces is considered a central process in the infection onset. Although Candida glabrata is frequently coisolated with C. albicans, its role in DS pathogenesis is unknown. We show here, using a contemporary rat model that employed a patented intraoral denture system, that C. glabrata established stable colonization on the denture/palate. However, in contrast to C. albicans inoculated rats, rats inoculated with C. glabrata exhibited minimal changes in weight gain or palatal tissue damage. Likewise, coinoculation with the two *Candida* species resulted in no exacerbation of C. albicans-induced DS pathology. Together, our findings indicate that C. glabrata has no inducing/enhancing role in DS pathogenesis.

## Abstract (precis)

Many denture wearers suffer from *Candida*-associated denture stomatitis (DS), a fungal infection of the hard palate in contact with dentures. Biofilm formation by Candida albicans on denture/palate surfaces is considered a central process in the infection onset. Although Candida glabrata is frequently coisolated with C. albicans, its role in DS pathogenesis is unknown. We show here, using a contemporary rat model that employed a patented intraoral denture system, that C. glabrata established stable colonization on the denture/palate. However, in contrast to C. albicans inoculated rats, rats inoculated with C. glabrata exhibited minimal changes in weight gain or palatal tissue damage. Likewise, coinoculation with the two *Candida* species resulted in no exacerbation of C. albicans-induced DS pathology. Together, our findings indicate that C. glabrata has no inducing/enhancing role in DS pathogenesis.

## INTRODUCTION

Denture stomatitis (DS) is an inflammatory fungal infection, presenting primarily as inflammation of oral mucosa beneath maxillary dentures (1–7). DS is by far the most common form of oral candidiasis, affecting approximately 70% of otherwise healthy denture wearers (8). DS is predominantly caused by Candida albicans, a dimorphic fungus that readily colonizes and forms biofilms on denture materials; however, non-*albicans Candida* species can also be associated with infection (9, 10). Candida glabrata is the second most common isolate, and up to 50% of patient samples contain more than one species of *Candida*, very often a combination of C. albicans and C. glabrata (3, 11–13). Manifestations of *Candida-*associated DS can range from being painless and asymptomatic to severe, involving erythematous and edematous palatal mucosa, painful inflammation, papillary hyperplasia (small pebble-like sores), and petechial hemorrhage (pinpoint bleeding) (14, 15). DS can have a negative impact on the quality of life of those affected, with high recurrence rates despite treatment with antifungal therapy (13, 16–21). Chronic DS infection could lead to seeding of the gastrointestinal tract, which serves as a major portal for systemic infection in immunosuppressed or hospitalized patients. Despite its high prevalence, the role of fungal virulence factors in the pathogenesis of DS has not been well defined.

Previous studies using an established rat model of DS showed a requirement for *Candida* biofilm formation on both palatal epithelium and denture surfaces in the initiation of infection and identified regulators of fungal morphogenesis (Efg1) and biofilm formation (Bcr1) as key players in DS pathogenesis (22). While C. glabrata, unlike C. albicans, does not undergo morphogenesis and thus is considered less virulent, both *Candida* species are frequently coisolated in mucosal candidiasis, including DS (9, 10, 23–25). Although single-species infection by C. glabrata alone is relatively rare, oral infections involving C. glabrata have shown an increasing trend over the past decade, especially in cancer patients, denture wearers, or those receiving prolonged antibiotic, steroid, or head and neck radiation therapies (10, 26–29). In addition, since C. glabrata displays significant resistance to azole antifungal drugs (23, 30–32), successful treatment of DS is likely challenging in cases of coinfection by both *Candida* species.

Despite its presence and ability to establish infection in animal models of oropharyngeal candidiasis (OPC) (33, 34), a pathogenic role for C. glabrata in DS remains unknown. In terms of adherence to biotic/abiotic surfaces, biofilm formation, and host tissue invasion, C. albicans has a major advantage over C. glabrata by its ability to transition from yeast to hyphae. In addition, C. albicans hyphal adhesins, such as agglutinin-like sequence (ALS) proteins and hyphal wall protein 1 (HWP1), also play an important role as binding sites for C. glabrata and other microorganisms, including Staphylococcus aureus (33, 35–39). C. glabrata virulence, on the other hand, likely involves cell wall proteins expressed independent of its morphology (35, 40–42). It is possible that cocolonization by C. glabrata with C. albicans may have additive impacts on virulence and pathogenicity compared to that by either species alone.

Using an established rat model of DS with a contemporary rodent denture system, we sought to determine whether C. glabrata alone or in combination with C. albicans establishes colonization and/or causes/enhances palatal tissue damage and inflammation.

## RESULTS

### C. glabrata establishes consistent colonization on dentures and palate tissues *in vivo*.

**FIG 1:**
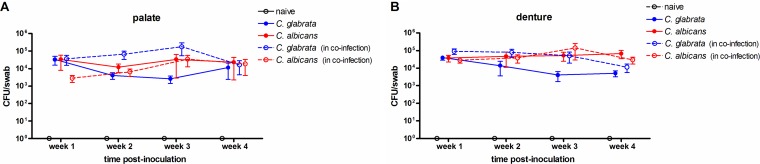
Fungal burden on dentures and palate tissues in rats inoculated with C. albicans and/or C. glabrata. Rats fitted with dentures were inoculated 3 times at 3-day intervals with 1 × 10^9^ CFU C. albicans, C. glabrata, or both species together (5 × 10^8^ CFU each). Swab samples of the palate (A) and denture (B) were collected weekly for a period of 4 weeks postinoculation. Fungal burden was assessed from overnight cultures of swab suspension fluid from the removable denture and associated palate tissue. Figures represent cumulative results from 2 independent experiments with 2 to 5 animals per group. Data were analyzed using repeated measures ANOVA (longitudinal data for each group) and one-way ANOVA (individual time points between groups) followed by the unpaired Student’s *t* test (experimental versus control groups at individual time points).

Rats installed with the denture system were inoculated with C. glabrata or C. albicans individually or the two species together and monitored longitudinally for a 4-week period. Fungal burden measured by swab collection demonstrated a consistent colonization with C. glabrata alone on the palate (Fig. 1A) and denture (Fig. 1B), similar to that with C. albicans. Coinoculation with the two *Candida* species resulted in a marked, but not statistically significant, increase in C. glabrata fungal burden (10- to 100-fold on dentures and palates at 2 to 3 weeks postinoculation). Levels of C. albicans were unaffected by coinoculation with C. glabrata.

### C. glabrata has no inducing or enhancing effects on C. albicans virulence.

**FIG 2:**
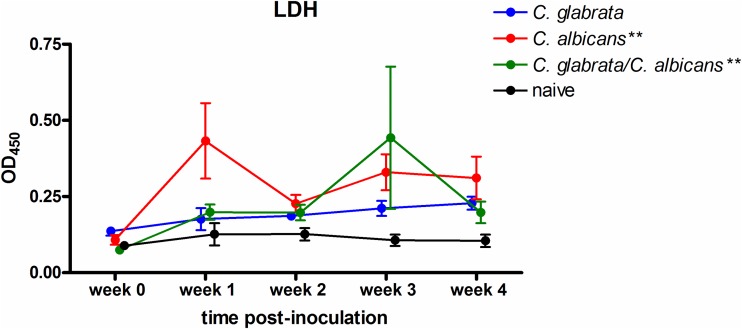
Palatal tissue damage over time in rats inoculated with C. albicans and/or C. glabrata. Rats fitted with dentures were inoculated 3 times at 3-day intervals with 1 × 10^9^ CFU C. albicans, C. glabrata, or both species together (5 × 10^8^ CFU each). Swab samples of the palate over the removable denture portion were collected weekly for a period of 4 weeks postinoculation. Swab suspension fluid was tested for LDH levels. Figure represents cumulative data from 2 independent experiments with 2 to 5 rats per group. Data were longitudinally analyzed by repeated measures ANOVA (significance indicated on graph legend) and comparatively analyzed by one-way ANOVA (individual time points between groups) followed by the unpaired Student's *t* test at specific time points. **, *P < *0.01.

**FIG 3:**
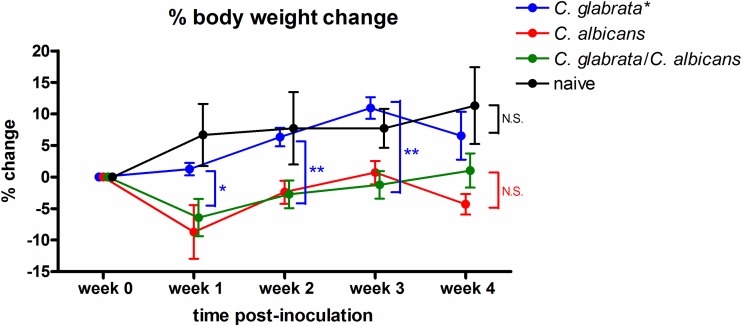
Body weight change over time in rats inoculated with C. albicans and/or C. glabrata. Rats fitted with dentures were inoculated 3 times at 3-day intervals with 1 × 10^9^ CFU C. albicans, C. glabrata, or both species together (5 × 10^8^ CFU each). Rats were weighed weekly for a period of 4 weeks postinoculation to assess the percent weight change (% weight change = [weight at time point/weight at week 0 prior to inoculation] × 100). Figure represents cumulative data from 2 independent experiments with 2 to 5 rats per group. Data were longitudinally analyzed by repeated measures ANOVA (significance indicated on graph legend) and comparatively analyzed by one-way ANOVA (individual time points between groups) followed by the unpaired Student’s *t* test at specific time points (significance indicated on data points). *, *P < *0.05; **, *P < *0.01; N.S., not significant.

Inoculated rats were evaluated for levels of LDH release by the palate, an indicator of tissue damage. Repeated measures analysis indicated that animals inoculated with C. albicans, alone or together with C. glabrata, exhibited significant modulation in levels of lactate dehydrogenase (LDH) over the course of infection (*P* = 0.003 and *P* = 0.002, respectively) (Fig. 2). In contrast, inoculation with C. glabrata alone induced minimal palatal LDH release with no apparent change under a consistent state of colonization. An indirect measure of virulence during infection is stunted weight gain over time, indicating a sign of DS-related discomfort in eating due to tissue damage in the oral cavity. Consistent with the lack of palatal tissue damage, colonization by C. glabrata alone resulted in normal weight gain comparable to that by naive animals over the 4 week period (Fig. 3). Conversely, animals inoculated with C. albicans alone or together with C. glabrata exhibited stunted weight gain (Fig. 3).

### C. glabrata does not promote inflammation.

**FIG 4:**
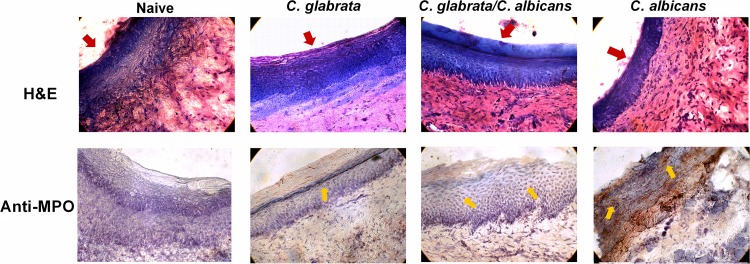
Histological analysis of palatal inflammation in rats inoculated with C. albicans and/or C. glabrata. Rats fitted with dentures were inoculated 3 times at 3-day intervals with 1 × 10^9^ CFU C. albicans, C. glabrata, or both species together (5 × 10^8^ CFU each). Palate tissue was harvested at 4 weeks postinoculation. Frozen tissue sections were stained with hematoxylin and eosin (H&E) for histopathological analysis or with anti-myeloperoxidase (MPO, brown-red) or isotype control (mouse IgG1) antibodies. Red arrows indicate the apical surface of the palate epithelium. Yellow arrows represent cells positively stained for MPO. Figure shows a representative result of 2 independent experiments. Magnification, ×400.

Palate tissues from inoculated rats at 4 weeks postinoculation were examined for evidence of inflammation. Histological analysis of palatal mucosa of rats inoculated with C. glabrata alone revealed few or no cellular infiltrates in lamina propria, with intact epithelial layers similar to naive tissues (Fig. 4, hematoxylin and eosin [H&E]). In contrast, palates from rats inoculated with C. albicans alone or together with C. glabrata demonstrated copious amounts of cellular infiltration as well as epithelial thinning and sloughing. Finally, the expression of the inflammatory marker myeloperoxidase (MPO) was markedly elevated by C. albicans colonization alone compared to that by C. glabrata colonization alone, with the combination of the two species showing moderate expression (Fig. 4, anti-MPO).

## DISCUSSION

In the present study using the contemporary rodent denture system, we demonstrated that C. glabrata has the ability to establish consistent colonization on both denture surfaces and palate tissues. C. glabrata is typically difficult to establish consistent colonization in experimental model systems involving biotic surfaces, presumably due to the lack of morphologic transition to hyphae as a virulence factor. For example, C. glabrata alone showed poor colonization on oral or vaginal reconstituted human epithelium (RHE) *in vitro* (35, 43, 44). *In vivo* models of murine oropharyngeal candidiasis (OPC) and vulvovaginal candidiasis (VVC) require corticosteroid-induced immunosuppression and a streptozotocin-induced diabetic state, respectively, to achieve consistent colonization (33, 34, 45). In the present DS model using immunocompetent rats, however, dentures appeared to serve as a stable reservoir for C. glabrata to sustain colonization. Indeed, C. glabrata is capable of growing on a variety of abiotic surfaces (34, 46, 47). The trend toward increased C. glabrata burden during cocolonization with C. albicans is consistent with recent evidence showing enhanced colonization by C. glabrata in a mouse OPC model following coinoculation with C. albicans (33). However, the lack of any statistically significant increase is more in line with studies reporting no changes in C. glabrata burden between mono- and cocolonization (34, 45). Hence, the observation is likely a minor attribute overall and does not appear to be suggestive of a synergistic outcome.

Biofilm formation by C. albicans has been exhaustively studied *in vitro* and *in vivo*, where hyphae provide scaffold structures that are essential for developing robust biofilms (22, 48–52). Furthermore, there is increasing evidence demonstrating that microorganisms preferentially bind to C. albicans hyphae in a polymicrobial environment (37, 39). This is presumably due to the fact that fungal adhesins are abundantly expressed on hyphal cell walls (33, 35–37, 53–55). Adherence to the hyphal surface and growth within biofilms are advantageous to many planktonic microbes in which the fungal polysaccharide extracellular matrix can provide protection from host defense and resistance to environmental stress and antimicrobials (56, 57). Interestingly, recent studies showed that despite its ability to colonize murine mucosal surfaces, colonization with C. glabrata alone did not result in appreciable biofilm formation on oral and vaginal epithelia (33, 45). This suggests that robust biofilm formation is not required for the survival of C. glabrata at mucosal sites. Although biofilms were not evaluated in our present study, we expect biofilm growth to be minimal on both palate mucosa and dentures in the absence of C. albicans. Support for this comes from our previous finding that hypha-deficient mutant strains of C. albicans failed to form mature biofilms despite sustained colonization (22). We hypothesize that the stable colonization of the palatal mucosa by C. glabrata or hypha-deficient C. albicans mutants is facilitated by the denture that serves as an adherence catalyst and feeder system for the mucosal tissue.

Contrary to its vigorous adhesion and colonization capacity, our results indicated that C. glabrata alone was not competent to cause a similar pathology observed in C. albicans-associated DS (tissue damage, weight loss, or palate inflammation) nor could it enhance C. albicans pathogenicity under coinoculated conditions. The lack of a pathogenic role for C. glabrata in monospecies colonization appears to be a common feature in several *in vitro* and *in vivo* models. Studies using oral epithelial cell culture showed no notable increase in proinflammatory cytokine production in response to C. glabrata alone (58, 59). Similarly, recent reports from both mouse OPC and VVC studies indicated that C. glabrata monoinfection resulted in only mild weight loss (OPC) and vaginal inflammation (VVC) (33, 45). Hence, our model, as well as others, has not been able to provide any clear evidence for a pathogenic role for C. glabrata monospecies infection at mucosal sites. It is possible, however, that C. glabrata monoinfections result in a more appreciable pathology in denture wearers under immunocompromising conditions (e.g., use of chemotherapies, prolonged antibiotics, advanced age).

The lack of any enhanced pathology under coinoculated conditions was surprising considering that coinoculation resulted in fungal burden (i.e., biomass) that was virtually doubled on both palate and dentures despite the reduced inoculum for each species (5 × 10^8^ for a total of 1 × 10^9^). In fact, one inflammatory marker, MPO, was actually decreased under coinoculated conditions. This result is likely due to the fact that DS occurs in immunocompetent subjects, both clinically and in our experimental model using immunocompetent rats. In agreement with this, studies in an immunocompetent mouse model of VVC (45), which resulted in a similar additive effect in fungal burden under coinoculated conditions, showed no changes in inflammatory response/tissue damage. On the other hand, studies using an immunosuppressed mouse OPC model (33) showed increased tissue damage and invasion during coinfection. Similarly, *in vitro* studies using a 3-dimensional (3-D) human oral mucosa model (60) or oral RHE model (43), which do not include immune cells, demonstrated C. glabrata strain-dependent effects on promoting tissue damage and invasion, even in the context of coinfection with C. albicans (43). While it is possible that the results in the DS model were strain dependent, the C. glabrata isolate chosen was based on its strong mucosal colonization capacity (45) and use in other model systems (VVC and intra-abdominal infection) (45, 61). Hence, while the isolate was not an oral isolate, it appeared representative for experimental models. Moreover, more recent studies in the intra-abdominal model using an oral C. glabrata isolate in parallel with the vaginal isolate yielded similar results (M. C. Noverr, unpublished observations), further supporting that strain-dependent attributes of C. glabrata pathogenicity in the DS model were unlikely. Additionally, in the OPC model, intimate binding of C. glabrata with C. albicans hyphae was observed, indicating that C. glabrata possibly exploits C. albicans to establish colonization and gain invasion into the oral epithelium under immunocompromised conditions (33, 43). In the VVC model, coinoculation with C. glabrata and C. albicans displayed a more interspersed presence throughout the tissue, with little interaction or colocalization, suggesting that the two species exist independent of each other (45). Therefore, interspecies interactions may also play pathogenic roles in OPC versus VVC. Because C. albicans rarely invades the hard palate, the likelihood that the two species would interact such to exploit each other in DS is low. Taken together, these arguments support the interpretation that there is no apparent contribution of C. glabrata in C. albicans-mediated DS pathogenesis.

Despite these results, a pathogenic potential of C. glabrata should not be underestimated due to its inherent resistance to azole compounds. Inadequate diagnosis and treatment of seemingly noninvasive C. glabrata infections could lead to more severe yet underreported cases of C. glabrata*-*associated candidiasis (e.g., fungal otitis, candidemia, candiduria) (62–67), which could potentially be a life-threatening condition if not treated in a timely manner. There is also the issue of microbial access to the gastrointestinal tract, where a continuous gastrointestinal exposure to *Candida* originating from denture biofilms could have a detrimental effect in denture wearers under immunocompromising conditions or those with advanced age who are at risk for immunosuppression. Indeed, patients with chronic DS have increased *Candida* carriage in the gastrointestinal tract, with similar species isolated from the oral cavity and feces (68). We also observed both C. albicans and C. glabrata in feces of inoculated mice, albeit in lower numbers than in the oral cavity (data not shown). As such, the rodent denture system represents an excellent model to further investigate these important pathogenesis questions along the entire oro-gastrointestinal tract.

## MATERIALS AND METHODS

### Animals.

Male CD hairless rats (7 weeks old) were purchased from Charles River Laboratories (Willington, MA). All rats were maintained in an AAALAC-accredited animal facility at Louisiana State University Health Sciences Center (LSUHSC) under a protocol approved by LSUHSC Institutional Animal Care and Use Committee. The animals were weaned onto gel diet A76 (ClearH2O, Westbrook, ME) and acclimated for at least 1 week prior to denture installation. The animals were maintained on the gel diet for the remainder of the study to minimize the accumulation of food debris on the denture.

### *Candida* species strains.

C. albicans strain DAY185, a prototrophic derivative of SC5314, was a gift from Aaron Mitchell (Carnegie Melon University, Pittsburgh, PA). C. glabrata strain LF 574.92 was provided by Jack Sobel (Wayne State University, Detroit, MI). Both *Candida* strains were grown in yeast extract-peptone-dextrose (YPD) broth for 18 h at 30°C with shaking at 200 rpm to reach a stationary-phase culture. Following incubation, the culture was washed 3 times in sterile phosphate-buffered saline (PBS) and enumerated on a hemocytometer using trypan blue dye.

### Rat denture stomatitis model.

Each rat was housed separately in an individual cage throughout the study period and handled according to institutionally recommended guidelines. A custom-fitted rodent denture system, consisting of fixed and removable portions, was employed (patent 8753113) (69, 70). For custom fitting, impressions of the palate were taken from individual rats using light-body VPS impression material (Aquasil Ultra LC; Dentsply Caulk). Impressions were used to produce stone mold templates for the fabrication of the fixed and removable denture components. For installation, rats were anesthetized by intraperitoneal injection with 90 mg/kg ketamine plus 10 mg/kg xylazine and remained sedated for at least 1 h to complete the installation process. The fixed portion of the denture containing nickel magnets was anchored to the rear molars by orthodontic ligature wires. The removable portion embedded with an aluminum rod was attached to the fixed portion via the nickel magnets and fitted over the anterior palate. The removable portion can easily be detached for sampling and replaced, which allows for longitudinal analyses. The rats installed with the dentures were given an additional acclimation period to ensure normal food and water intake. For inoculation, rats were anesthetized by isoflurane inhalation and inoculated by applying an oral gel (PBS semisolidified with 5% carboxymethylcellulose; Sigma) containing C. albicans (1 × 10^9^), C. glabrata (1 × 10^9^), or the two species together (5 × 10^8^ each) on the palate beneath the removable denture. The rats remained anesthetized until the removable denture was securely reinstalled with the gel inoculum in place. Inoculation was performed a total of 3 times separated by 3-day intervals, and rats were monitored weekly over a 4-week period for oral outcome parameters, signs of distress, and weight changes. Control animals (naive) were rats with dentures installed and given gel alone.

### Quantification of microbial burden.

To assess fungal burden on the denture and palate tissue, rats were anesthetized by isoflurane inhalation, and the removable portion of the denture was detached using sterile forceps. The intaglio surface of the denture and the palate were swabbed with individual sterile cotton tipped applicators. Swabbing was performed by gently sliding the cotton applicator on the denture surface or the hard palate along the ridges of the rugae. Swab tips were immersed in 200 μl PBS and vigorously mixed. To assess fungal burden, serial dilutions of the swab supernatants were cultured on Sabouraud dextrose agar (BD Diagnostics) for 24 h at 37°C. CFUs were enumerated and expressed as CFU/swab.

### Assessment of palatal tissue damage.

To determine tissue damage, the levels of lactose dehydrogenase (LDH) release in palates were measured by an LDH assay kit as per the manufacturer’s instructions (Abcam). The activity of LDH in the supernatants of palate swab suspensions was measured with a colorimetric probe. The absorbance was read at a wavelength of 450 nm using a Multiskan Ascent microplate photometer (Labsystems). The results were expressed as the optical density at 450 nm (OD_450_).

### Microscopic evaluation of palatal tissues.

Palate tissue was excised from euthanized rats at 4 weeks postinoculation. Tissue specimens were placed in Tissue-Tek cryomolds (Miles Corp.) containing optimum cutting temperature (OCT) medium (Sakura Finetek) and stored at −80°C. Frozen tissue was sectioned (6 μm) and collected on glass slides. The slides were either processed for a hematoxylin and eosin (H&E) staining for histology or fixed in ice-cold acetone for 5 min and stored at −20°C until use. For immunohistochemical analysis, tissue sections were hydrated in PBS and processed using a cell and tissue staining kit (horseradish peroxidase [HRP]-3-amino-9-ethylcarbazole; R&D Systems). Briefly, tissue slides were blocked with peroxidase, goat serum, avidin, and biotin blocking buffers and then incubated with monoclonal mouse anti-rat myeloperoxidase (MPO) antibody (10 μg/ml; R&D Systems) or isotype control antibody (mouse IgG1) overnight at 4°C. The slides were washed and incubated with biotinylated anti-mouse IgG antibodies for 1 h at room temperature followed by streptavidin-HRP for 30 min. The slides were then reacted with AEC chromogen substrate, counterstained with CAT hematoxylin (Biocare Medical), and preserved in aqueous mounting medium (R&D Systems). Images were captured at ×400 magnification.

### Statistics.

All experiments included groups of 2 to 5 rats and were repeated twice. Longitudinal data of fungal burden, LDH levels, and percent weight change were analyzed by repeated measures analysis of variance (ANOVA) to identify changes over time within each group. Data were further analyzed using a one-way ANOVA followed by the Tukey’s *post hoc* multiple-comparison test to identify differences between groups at specific time points. The Student’s *t* test was used to compare the experimental groups to relevant control groups. Statistical significance was defined at a confidence level where *P* was <0.05. All statistical analyses were performed using Prism software (Graph Pad).

## References

[1] Budtz-Jorgensen E, Stenderup A, Grabowski M 1975 An epidemiologic study of yeasts in elderly denture wearers. Community Dent Oral Epidemiol 3:115–119. doi:10.1111/j.1600-0528.1975.tb00291.x. 1056815

[2] Arendorf TM, Walker DM 1987 Denture stomatitis: a review. J Oral Rehabil 14:217–227. doi:10.1111/j.1365-2842.1987.tb00713.x. 3298586

[3] Cumming CG, Wight C, Blackwell CL, Wray D 1990 Denture stomatitis in the elderly. Oral Microbiol Immunol 5:82–85. doi:10.1111/j.1399-302X.1990.tb00232.x. 2087353

[4] Budtz-Jorgensen E 1981 Oral mucosal lesions associated with the wearing of removable dentures. J Oral Pathol Med 10:65–80. doi:10.1111/j.1600-0714.1981.tb01251.x. 6792333

[5] Pires FR, Santos EB, Bonan PR, De Almeida OP, Lopes MA 2002 Denture stomatitis and salivary *Candida* in Brazilian edentulous patients. J Oral Rehabil 29:1115–1119. doi:10.1046/j.1365-2842.2002.00947.x. 12453267

[6] Shulman JD, Rivera-Hidalgo F, Beach MM 2005 Risk factors associated with denture stomatitis in the United States. J Oral Pathol Med 34:340–346. doi:10.1111/j.1600-0714.2005.00287.x. 15946181

[7] Zissis A, Yannikakis S, Harrison A 2006 Comparison of denture stomatitis prevalence in 2 population groups. Int J Prosthodont 19:621–625. 17165305

[8] Gendreau L, Loewy ZG 2011 Epidemiology and etiology of denture stomatitis. J Prosthodont 20:251–260. doi:10.1111/j.1532-849X.2011.00698.x. 21463383

[9] Pereira CA, Toledo BC, Santos CT, Pereira Costa AC, Back-Brito GN, Kaminagakura E, Jorge AO 2013 Opportunistic microorganisms in individuals with lesions of denture stomatitis. Diagn Microbiol Infect Dis 76:419–424. doi:10.1016/j.diagmicrobio.2013.05.001. 23747028

[10] Redding SW, Kirkpatrick WR, Coco BJ, Sadkowski L, Fothergill AW, Rinaldi MG, Eng TY, Patterson TF 2002 *Candida glabrata* oropharyngeal candidiasis in patients receiving radiation treatment for head and neck cancer. J Clin Microbiol 40:1879–1881. doi:10.1128/JCM.40.5.1879-1881.2002. 11980984 PMC130928

[11] Dorocka-Bobkowska B, Konopka K 2007 Susceptibility of *Candida* isolates from denture-related stomatitis to antifungal agents *in vitro*. Int J Prosthodont 20:504–506. 17944341

[12] Zomorodian K, Haghighi NN, Rajaee N, Pakshir K, Tarazooie B, Vojdani M, Sedaghat F, Vosoghi M 2011 Assessment of *Candida* species colonization and denture-related stomatitis in complete denture wearers. Med Mycol 49:208–211. doi:10.3109/13693786.2010.507605. 20795762

[13] Vanden Abbeele A, de Meel H, Ahariz M, Perraudin JP, Beyer I, Courtois P 2008 Denture contamination by yeasts in the elderly. Gerodontology 25:222–228. doi:10.1111/j.1741-2358.2007.00247.x. 18665849

[14] Webb BC, Thomas CJ, Willcox MD, Harty DW, Knox KW 1998 *Candida*-associated denture stomatitis. Aetiology and management: a review. Part 3. Treatment of oral candidosis. Aust Dent J 43:244–249. doi:10.1111/j.1834-7819.1998.tb00172.x. 9775471

[15] Scully C, Felix DH 2005 Oral medicine–update for the dental practitioner: red and pigmented lesions. Br Dent J 199:639–645. doi:10.1038/sj.bdj.4813017. 16311559

[16] Budtz-Jorgensen E, Kelstrup J, Poulsen S 1983 Reduction of formation of denture plaque by a protease (Alcalase). Acta Odontol Scand 41:93–98. doi:10.3109/00016358309162308. 6349232

[17] Bergendal T, Holmberg K 1982 Studies of *Candida* serology in denture stomatitis patients. Scand J Dent Res 90:315–322. 6957970 10.1111/j.1600-0722.1982.tb00743.x

[18] Lombardi T, Budtz-Jörgensen E 1993 Treatment of denture-induced stomatitis: a review. Eur J Prosthodont Restor Dent 2:17–22. 8180613

[19] Budtz-Jorgensen E, Holmstrup P, Krogh P 1988 Fluconazole in the treatment of *Candida*-associated denture stomatitis. Antimicrob Agents Chemother 32:1859–1863. doi:10.1128/AAC.32.12.1859. 2854455 PMC176033

[20] Hilgert JB, Giordani JM, de Souza RF, Wendland EM, D'Avila OP, Hugo FN 2016 Interventions for the management of denture stomatitis: a systematic review and meta-analysis. J Am Geriatr Soc 64:2539–2545. doi:10.1111/jgs.14399. 27889906

[21] Lima JF, Maciel JG, Arrais CA, Porto VC, Urban VM, Neppelenbroek KH 2016 Effect of incorporating antifungals on the water sorption and solubility of interim resilient liners for denture base relining. J Prosthet Dent 115:611–616. doi:10.1016/j.prosdent.2015.09.029. 26794705

[22] Yano J, Yu A, Fidel PL Jr, Noverr MC 2016 Transcription factors Efg1 and Bcr1 regulate biofilm formation and virulence during *Candida albicans*-associated denture stomatitis. PLoS One 11:e0159692. doi:10.1371/journal.pone.0159692. 27453977 PMC4959791

[23] Fidel PL Jr, Vazquez JA, Sobel JD 1999 *Candida glabrata*: review of epidemiology, pathogenesis, and clinical disease with comparison to *C. albicans*. Clin Microbiol Rev 12:80–96. doi:10.1128/CMR.12.1.80. 9880475 PMC88907

[24] Redding SW, Zellars RC, Kirkpatrick WR, McAtee RK, Caceres MA, Fothergill AW, Lopez-Ribot JL, Bailey CW, Rinaldi MG, Patterson TF 1999 Epidemiology of oropharyngeal *Candida* colonization and infection in patients receiving radiation for head and neck cancer. J Clin Microbiol 37:3896–3900. 10565903 10.1128/jcm.37.12.3896-3900.1999 PMC85839

[25] Redding SW 2001 The role of yeasts other than *Candida albicans* in oropharyngeal candidiasis. Curr Opin Infect Dis 14:673–677. doi:10.1097/00001432-200112000-00002. 11964883

[26] Dongari-Bagtzoglou A, Dwivedi P, Ioannidou E, Shaqman M, Hull D, Burleson J 2009 Oral *Candida* infection and colonization in solid organ transplant recipients. Oral Microbiol Immunol 24:249–254. doi:10.1111/j.1399-302X.2009.00505.x. 19416456 PMC2699610

[27] Belazi M, Velegraki A, Koussidou-Eremondi T, Andreadis D, Hini S, Arsenis G, Eliopoulou C, Destouni E, Antoniades D 2004 Oral *Candida* isolates in patients undergoing radiotherapy for head and neck cancer: prevalence, azole susceptibility profiles and response to antifungal treatment. Oral Microbiol Immunol 19:347–351. doi:10.1111/j.1399-302x.2004.00165.x. 15491459

[28] Vazquez JA 1999 Options for the management of mucosal candidiasis in patients with AIDS and HIV infection. Pharmacotherapy 19:76–87. doi:10.1592/phco.19.1.76.30509. 9917080

[29] Coco BJ, Bagg J, Cross LJ, Jose A, Cross J, Ramage G 2008 Mixed *Candida albicans* and *Candida glabrata* populations associated with the pathogenesis of denture stomatitis. Oral Microbiol Immunol 23:377–383. doi:10.1111/j.1399-302X.2008.00439.x. 18793360

[30] Dorocka-Bobkowska B, Konopka K, Düzgüneş N 2003 Influence of antifungal polyenes on the adhesion of *Candida albicans* and *Candida glabrata* to human epithelial cells *in vitro*. Arch Oral Biol 48:805–814. doi:10.1016/S0003-9969(03)00174-2. 14596870

[31] Colombo AL, Junior JNA, Guinea J 2017 Emerging multidrug-resistant *Candida* species. Curr Opin Infect Dis 30:528–538. doi:10.1097/QCO.0000000000000411. 29095200

[32] Sobel JD 2000 Management of infections caused by *Candida glabrata*. Curr Infect Dis Rep 2:424–428. doi:10.1007/s11908-000-0069-x. 11095887

[33] Tati S, Davidow P, McCall A, Hwang-Wong E, Rojas IG, Cormack B, Edgerton M 2016 *Candida glabrata* binding to *Candida albicans* hyphae enables its development in oropharyngeal candidiasis. PLoS Pathog 12:e1005522. doi:10.1371/journal.ppat.1005522. 27029023 PMC4814137

[34] Rossoni RD, Barbosa JO, Vilela SF, dos Santos JD, de Barros PP, Prata MC, Anbinder AL, Fuchs BB, Jorge AO, Mylonakis E, Junqueira JC 2015 Competitive interactions between *C. albicans*, *C. glabrata* and *C. krusei* during biofilm formation and development of experimental candidiasis. PLoS One 10:e0131700. doi:10.1371/journal.pone.0131700. 26146832 PMC4493022

[35] Alves CT, Wei XQ, Silva S, Azeredo J, Henriques M, Williams DW 2014 *Candida albicans* promotes invasion and colonisation of *Candida glabrata* in a reconstituted human vaginal epithelium. J Infect 69:396–407. doi:10.1016/j.jinf.2014.06.002. 24924556

[36] Silverman RJ, Nobbs AH, Vickerman MM, Barbour ME, Jenkinson HF 2010 Interaction of *Candida albicans* cell wall Als3 protein with *Streptococcus gordonii* SspB adhesin promotes development of mixed-species communities. Infect Immun 78:4644–4652. doi:10.1128/IAI.00685-10. 20805332 PMC2976310

[37] Peters BM, Ovchinnikova ES, Krom BP, Schlecht LM, Zhou H, Hoyer LL, Busscher HJ, van der Mei HC, Jabra-Rizk MA, Shirtliff ME 2012 *Staphylococcus aureus* adherence to *Candida albicans* hyphae is mediated by the hyphal adhesin Als3p. Microbiology 158:2975–2986. doi:10.1099/mic.0.062109-0. 22918893 PMC4083660

[38] Mear JB, Kipnis E, Faure E, Dessein R, Schurtz G, Faure K, Guery B 2013 *Candida albicans* and *Pseudomonas aeruginosa* interactions: more than an opportunistic criminal association? Med Mal Infect 43:146–151. doi:10.1016/j.medmal.2013.02.005. 23622953

[39] Bamford CV, Nobbs AH, Barbour ME, Lamont RJ, Jenkinson HF 2015 Functional regions of *Candida albicans* hyphal cell wall protein Als3 that determine interaction with the oral bacterium *Streptococcus gordonii*. Microbiology 161:18–29. doi:10.1099/mic.0.083378-0. 25332379 PMC4274786

[40] Willaert RG 2018 Adhesins of yeasts: protein structure and interactions. J Fungi (Basel) 4:E119. doi:10.3390/jof4040119. 30373267 PMC6308950

[41] Castano I, Pan SJ, Zupancic M, Hennequin C, Dujon B, Cormack BP 2005 Telomere length control and transcriptional regulation of subtelomeric adhesins in *Candida glabrata*. Mol Microbiol 55:1246–1258. doi:10.1111/j.1365-2958.2004.04465.x. 15686568

[42] Zupancic ML, Frieman M, Smith D, Alvarez RA, Cummings RD, Cormack BP 2008 Glycan microarray analysis of *Candida glabrata* adhesin ligand specificity. Mol Microbiol 68:547–559. doi:10.1111/j.1365-2958.2008.06184.x. 18394144

[43] Silva S, Henriques M, Hayes A, Oliveira R, Azeredo J, Williams DW 2011 *Candida glabrata* and *Candida albicans* co-infection of an *in vitro* oral epithelium. J Oral Pathol Med 40:421–427. doi:10.1111/j.1600-0714.2010.00981.x. 21158929

[44] Jayatilake JA, Samaranayake YH, Cheung LK, Samaranayake LP 2006 Quantitative evaluation of tissue invasion by wild type, hyphal and SAP mutants of *Candida albicans*, and non-albicans *Candida* species in reconstituted human oral epithelium. J Oral Pathol Med 35:484–491. doi:10.1111/j.1600-0714.2006.00435.x. 16918600

[45] Nash EE, Peters BM, Lilly EA, Noverr MC, Fidel PL Jr. 2016 A murine model of *Candida glabrata* vaginitis shows no evidence of an inflammatory immunopathogenic response. PLoS One 11:e0147969. doi:10.1371/journal.pone.0147969. 26807975 PMC4726552

[46] Persyn A, Rogiers O, Brock M, Vande Velde G, Lamkanfi M, Jacobsen ID, Himmelreich U, Lagrou K, Van Dijck P, Kucharikova S 2019 Monitoring of fluconazole and caspofungin activity against *in vivo Candida glabrata* biofilms by bioluminescence imaging. Antimicrob Agents Chemother 63:e01555-18. doi:10.1128/AAC.01555-1. 30420485 PMC6355587

[47] Estivill D, Arias A, Torres-Lana A, Carrillo-Muñoz AJ, Arévalo MP 2011 Biofilm formation by five species of *Candida* on three clinical materials. J Microbiol Methods 86:238–242. doi:10.1016/j.mimet.2011.05.019. 21664387

[48] Dongari-Bagtzoglou A, Kashleva H, Dwivedi P, Diaz P, Vasilakos J 2009 Characterization of mucosal *Candida albicans* biofilms. PLoS One 4:e7967. doi:10.1371/journal.pone.0007967. 19956771 PMC2776351

[49] Blankenship JR, Mitchell AP 2006 How to build a biofilm: a fungal perspective. Curr Opin Microbiol 9:588–594. doi:10.1016/j.mib.2006.10.003. 17055772

[50] Radford DR, Challacombe SJ, Walter JD 1999 Denture plaque and adherence of *Candida albicans* to denture-base materials *in vivo* and *in vitro*. Crit Rev Oral Biol Med 10:99–116. doi:10.1177/10454411990100010501. 10759429

[51] Nett JE, Marchillo K, Spiegel CA, Andes DR 2010 Development and validation of an *in vivo Candida albicans* biofilm denture model. Infect Immun 78:3650–3659. doi:10.1128/IAI.00480-10. 20605982 PMC2937450

[52] Harriott MM, Lilly EA, Rodriguez TE, Fidel PL, Noverr MC 2010 *Candida albicans* forms biofilms on the vaginal mucosa. Microbiology 156:3635–3644. doi:10.1099/mic.0.039354-0. 20705667 PMC3068702

[53] Dwivedi P, Thompson A, Xie Z, Kashleva H, Ganguly S, Mitchell AP, Dongari-Bagtzoglou A 2011 Role of Bcr1-activated genes Hwp1 and Hyr1 in *Candida albicans* oral mucosal biofilms and neutrophil evasion. PLoS One 6:e16218. doi:10.1371/journal.pone.0016218. 21283544 PMC3026825

[54] Nobile CJ, Andes DR, Nett JE, Smith FJ, Yue F, Phan QT, Edwards JE, Filler SG, Mitchell AP 2006 Critical role of Bcr1-dependent adhesins in *C. albicans* biofilm formation *in vitro* and *in vivo*. PLoS Pathog 2:e63. doi:10.1371/journal.ppat.0020063. 16839200 PMC1487173

[55] Nobile CJ, Nett JE, Andes DR, Mitchell AP 2006 Function of *Candida albicans* adhesin Hwp1 in biofilm formation. Eukaryot Cell 5:1604–1610. doi:10.1128/EC.00194-06. 17030992 PMC1595337

[56] Katragkou A, Kruhlak MJ, Simitsopoulou M, Chatzimoschou A, Taparkou A, Cotten CJ, Paliogianni F, Diza-Mataftsi E, Tsantali C, Walsh TJ, Roilides E 2010 Interactions between human phagocytes and *Candida albicans* biofilms alone and in combination with antifungal agents. J Infect Dis 201:1941–1949. doi:10.1086/652783. 20415537 PMC2911126

[57] Katragkou A, Simitsopoulou M, Chatzimoschou A, Georgiadou E, Walsh TJ, Roilides E 2011 Effects of interferon-gamma and granulocyte colony-stimulating factor on antifungal activity of human polymorphonuclear neutrophils against *Candida albicans* grown as biofilms or planktonic cells. Cytokine 55:330–334. doi:10.1016/j.cyto.2011.05.007. 21641233

[58] Li L, Dongari-Bagtzoglou A 2007 Oral epithelium-*Candida glabrata* interactions *in vitro*. Oral Microbiol Immunol 22:182–187. doi:10.1111/j.1399-302X.2007.00342.x. 17488444

[59] Schaller M, Mailhammer R, Grassl G, Sander CA, Hube B, Korting HC 2002 Infection of human oral epithelia with *Candida* species induces cytokine expression correlated to the degree of virulence. J Invest Dermatol 118:652–657. doi:10.1046/j.1523-1747.2002.01699.x. 11918712

[60] Li L, Kashleva H, Dongari-Bagtzoglou A 2007 Cytotoxic and cytokine-inducing properties of *Candida glabrata* in single and mixed oral infection models. Microb Pathog 42:138–147. doi:10.1016/j.micpath.2006.12.003. 17306958 PMC1973167

[61] Lilly EA, Ikeh M, Nash EE, Fidel PL Jr, Noverr MC 2018 Immune protection against lethal fungal-bacterial intra-abdominal infections. mBio 9:e01472-17. doi:10.1128/mBio.01472-17. 29339423 PMC5770546

[62] Pinkert H, Harper MB, Cooper T, Fleisher GR 1993 HIV-infected children in the pediatric emergency department. Pediatr Emerg Care 9:265–269. doi:10.1097/00006565-199310000-00002. 8247930

[63] Bae WK, Lee KS, Park JW, Bae EH, Ma SK, Kim NH, Choi KC, Shin JH, Cho HH, Cho YB, Kim SW 2007 A case of malignant otitis externa caused by *Candida glabrata* in a patient receiving haemodialysis. Scand J Infect Dis 39:370–372. doi:10.1080/00365540600978971. 17454907

[64] Berlanga GA, Machen GL, Lowry PS, Brust KB 2016 Management of a renal fungal bezoar caused by multidrug-resistant *Candida glabrata*. Proc (Bayl Univ Med Cent) 29:416–417. doi:10.1080/08998280.2016.11929493. 27695182 PMC5023304

[65] Shi R, Zhou Q, Fang R, Xiong X, Wang Q 2018 Severe acute pancreatitis with blood infection by *Candida glabrata* complicated severe agranulocytosis: a case report. BMC Infect Dis 18:706. doi:10.1186/s12879-018-3623-6. 30594147 PMC6310945

[66] Smyth J, Mullen CC, Jack L, Collier A, Bal AM 2018 Diabetes, malignancy and age as predictors of *Candida glabrata* bloodstream infection: a re-evaluation of the risk factors. J Mycol Med 28:547–550. doi:10.1016/j.mycmed.2018.05.004. 29803698

[67] Barchiesi F, Orsetti E, Mazzanti S, Trave F, Salvi A, Nitti C, Manso E 2017 Candidemia in the elderly: what does it change? PLoS One 12:e0176576. doi:10.1371/journal.pone.0176576. 28493896 PMC5426612

[68] Bergendal T, Holmberg K, Nord CE 1979 Yeast colonization in the oral cavity and feces in patients with denture stomatitis. Acta Odontol Scand 37:37–45. doi:10.3109/00016357909004683. 371327

[69] Lee H, Yu A, Johnson CC, Lilly EA, Noverr MC, Fidel PL Jr. 2011 Fabrication of a multi-applicable removable intraoral denture system for rodent research. J Oral Rehabil 38:686–690. doi:10.1111/j.1365-2842.2011.02206.x. 21323935 PMC3139747

[70] Johnson CC, Yu A, Lee H, Fidel PL Jr, Noverr MC 2012 Development of a contemporary animal model of *Candida albicans-*associated denture stomatitis using a novel intraoral denture system. Infect Immun 80:1736–1743. doi:10.1128/IAI.00019-12. 22392931 PMC3347462

